# Rapid Detection of Kaempferol Using Surface Molecularly Imprinted Mesoporous Molecular Sieves Embedded with Carbon Dots

**DOI:** 10.1155/2020/5819062

**Published:** 2020-07-10

**Authors:** Yu He, Junping Wang, Shuo Wang

**Affiliations:** ^1^State Key Laboratory of Food Nutrition and Safety, Tianjin University of Science and Technology, Tianjin 300457, China; ^2^Medical College, Nankai University, Tianjin 300350, China

## Abstract

This work demonstrates rapid sensing of kaempferol using active sensing material synthesized using the one-pot surface-imprinting synthesis method. This sensor consisted of molecularly imprinted polymer (MIP) consisting of mesoporous molecular sieves (SBA-15) loaded with carbon dots (CDs). Fourier transform infrared (FT-IR) spectroscopy confirmed successful incorporation of CDs onto the surface of imprinted mesoporous molecular sieves. Ordered hexagonal arrays of CDs@SBA-15@MIP mesopore structure were confirmed with transmission electron microscopy. Fluorescence intensity of CDs@SBA-15@MIP composites linearly correlated with kaempferol content in the 0.05–2 mg/L range. Detection limit was 14 *μ*g/L. MIPs were used for efficient detection of kaempferol in fruit and vegetable samples with recovery values from 80% to 112%. The method has high sensitivity, low cost, good selectivity, and many application potentials useful for research and development of flavonoid monomer presence in food.

## 1. Introduction

Kaempferol, a polyphenolic antioxidant present in vegetables and fruits, is a very beneficial dietary component because it is able to reduce risk of cancer and various chronic illnesses [1]. Epidemiological studies showed direct correlation between amount of consumed kaempferol and cancer reduction [2]. Kaempferol helps to fight cancer and chronic diseases by boosting antioxidant-based defense of the organisms against aggressive and cancer-causing free radicals, which, at molecular level, translates into kaempferol control of key factors of cellular signal transduction routes linked to apoptosis, inflammation, angiogenesis, and metastasis [3, 4].

Most common kaempferol detection methods are high-performance liquid chromatography (HPLC) combined with UV-detection, mass spectrometry, colorimetry, etc [5–10]. Chromatography is accurate for detection of single or multiple components. However, it requires complex and tedious preprocessing steps and expensive equipment. Colorimetric methods are fast and easy and are the most common detection methods. Yet, they only detect total flavonoid contents. A technique to specifically and selectively detect kaempferol in food sample as well as to distinguish kaempferol from other flavonoids is urgently needed to screen and describe food nutritional contents. Such technique also needs to be very sensitive to variety of kaempferol contents as well as to be simple, inexpensive, and convenient. Previous work has highlighted the excellent potential for the application of fluorescence spectroscopy to food safety evaluations; as such, an approach can be employed both effectively in research contexts and in assessments of food quality [11]. When assessing food quality, this approach can be used to monitor the egg freshness during storage, to explore the evolution of extra virgin olive oils under illumination, to detect aflatoxin and related secondary metabolites, and to measure total amino acids in herbicide-stressed oilseed rape leaves [12–15].

At present, nanotechnology has facilitated the development of sensors with improved sensitivity and selectivity, thus offering novel opportunities for substantial innovation [16]. Two materials, used in this work to develop such technique, are mesoporous molecular sieves and carbon dots (CDs). Mesoporous molecular sieves are excellent extraction materials because of their ordered structures, high surface areas, uniform and adjustable pore sizes, and outstanding chemical stability [17, 18]. CDs-based composites are relatively recently developed fluorescent materials, which demonstrate better photostability, brighter photoluminescence, better biocompatibility, and lower background noise in comparison to traditional organic dyes [19].

**Scheme 1:**
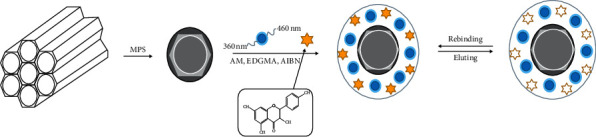
Preparation of CDs@SBA-15@MIP.

In this work, using these two materials, we created molecular imprinted polymers (MIPs) with the goal to obtain an effective and simple one-stage technique of material preparation for sensing. We created MIP based on mesoporous molecular sieve (SBA-15), which was loaded with CDs. Our main aim was to create a sensor capable to detect kaempferol accurately as well as with high sensitivity and selectively (Scheme 1). Surface molecular imprinting technique was used to obtain MIP composite. The resulting CDs@SBA-15@MIP composites consisted of highly ordered hexagonal mesopore arrays aligned as one-dimensional channels. At optimized conditions, linear correlation between sensor response and kaempferol concentration in the 0.05–2.0 mg/L range was observed. In this kaempferol concentration range, the sensor demonstrated rapid response time and excellent selectivity to distinguish kaempferol from its structural analogues. Fluorescence-based detection of kaempferol using CDs@SBA-15@MIPs as active sensing material demonstrates novel strategy for selective and sensitive kaempferol analysis in vegetable and fruits without need of bulky and expensive equipment such as liquid chromatography and mass spectrometry.

## 2. Materials and Methods

### 2.1. Materials

N-(*β*-aminoethyl)-*γ*-aminopropyl methyldimethoxysilane (AEAPMS), 3-methacryloxypropyltrimethoxysilane (MPS), and 2, 2-azobisisobutyronitrile (AIBN) were purchased from Tianjin Kermel Chemical Reagent (China). SBA-15 was obtained from Xfnano Reagents (Nanjing, China). Kaempferol was acquired from TCI Development (Shanghai, China). Citric acid (CA), acrylamide (AM), tetra-ethoxy-silane (TEOS), ethylene glycol di-methacrylate (EGDMA), and other chemicals were obtained from Sinopharm Chemical Reagent (Tianjin, China). Fruits and vegetables were purchased from a local grocery store.

### 2.2. Characterization

Fourier transform infrared (FT-IR) spectroscopy was performed using VECTOR-22 (Bruker, Germany). Fluorescence (FL) spectra were recorded using Thermo Scientific Lumina FL-4500 spectrometer (Thermo America, USA) with the 365 nm wavelength, 10 nm wide excitation, and emission slits as well as using 700 V photomultiplier tube voltage. Absorbance was measured using Thermo Scientific Evolution 300 UV-vis spectrophotometer (USA).

### 2.3. Carbon Dot Synthesis

CDs were obtained by a one-step reaction synthesis first implemented by Wang et al. [20]. For this purpose, 10 mL of AEAPMS was degassed by flushing the 100 mL three-necked flask with nitrogen gas for 5 minutes, after which the reaction solution was heated to 240°C and 0.5 g of CA was quickly added under constant vigorous stirring for 1 minute. After that, the system was allowed to cool naturally. Final CDs-containing products were collected by petroleum-ether-assisted precipitation performed 3 times. Samples were stored at 4°C until they were used and/or analyzed.

### 2.4. SBA-15-MPS Synthesis

SBA-15-MPS was fabricated using method reported by He et al. [21]. For this purpose, 500 mg of SBA-15 was mixed with 50 mL of toluene. Then, 10 mL of MPS was added, and the mixture was stirred at 55°C for 24 h under nitrogen gas atmosphere. Final solid product formed was centrifuged, rinsed with toluene and methanol, and then dried at 45°C in a vacuum furnace for 24 h.

### 2.5. Synthesis of CDs@SBA-15@MIPs and CDs@SBA-15@NIPs

0.28 mmol kaempferol and 1.55 mmol AM were dispersed in 25 ml of tetrahydrofuran/ethanol mixture (2.5 : 1 volume ratio) under constant stirring for 4 h at room temperature. The resulting suspension was named “mixture A.” 5.7 mmol of EGDMA, acting as a cross linker, 70 mg of AIBN initiator, and 0.1 g of SBA-15-MPS were dispersed in 25 mL of tetrahydrofuran/ethanol mixture (with 3 : 2 volume ratio). This suspension was named “mixture B.”

Mixture B was transferred into mixture A, and 20 *μ*L of CDs was added in A. The resulting solution was first stirred for 10 min and then flushed with nitrogen gas for 15 min. To allow polymerization to proceed, the reaction vessel was sealed and kept for 24 hours at 60°C in a water bath, after which the final product was washed with methanol/acetic acid mixture (with 9 : 1 volume ratio). To ensure complete removal of the template, discarded liquid was analyzed by UV-vis spectroscopy (Scheme 1). Thus, rinsing of the final product stopped when no template was detected in the decanted solution. Final CDs@SBA-15@MIPs were dried at 60°C in vacuum for 10 h. For comparison, CDs@SBA-15@NIP was synthesized following the same route as described above without addition of kaempferol.

### 2.6. Measurements of FL

To evaluate adsorption and selectivity of the synthesized materials, 1 mg CDs@SBA-15@MIP or CDs@SBA-15@NIP was thoroughly dispersed in 4 mL of kaempferol (and its analogues) at different concentrations. The solutions were shaken for 2 hours at room temperature, and FL intensity of each solution was measured before and after its reaction with kaempferol.

### 2.7. Sample Preparation

Fruits and vegetables were crushed in a mixer grinder. 1 g of each sample was then placed into a 50 mL disposable screw-capped polypropylene tube. Then, 25 ml of 80% ethanol was added for a 30 min extraction process performed in an ultrasonic bath. Extraction step was repeated two times. The obtained filtrate was then evaporated using a rotary evaporator. The resulting solid residue was dissolved in 2 mL of ethanol and filtered through a nylon micropore membrane with 0.45 *μ*m micropores. Samples were stored in a clean glass bottle until further characterization.

### 2.8. HPLC Analysis

Extracts were purified using C_18_ column to eliminate matrix interferences. Eluent obtained from the column was dried on a rotary evaporation, dissolved again in 2 mL of ethanol, and finally filtered using a nylon membrane with 0.45 *μ*m micropores for further HPLC-UV tests.

High-performance liquid chromatography (HPLC) coupled with UV was performed by using the LC-20AT (Shimadzu) chromatograph. Samples sizes were 20 *μ*L. Analysis was performed using a diode array detector (SPD-20A, Shimadzu) at 360 nm. Mixture of water and methanol at 2 : 3 volume ratio was used as a mobile phase, flow rate of which was of 1.0 mL/min.

## 3. Results and Discussion

### 3.1. Properties of Obtained CDs@SBA-15@MIP

**Figure 1:**
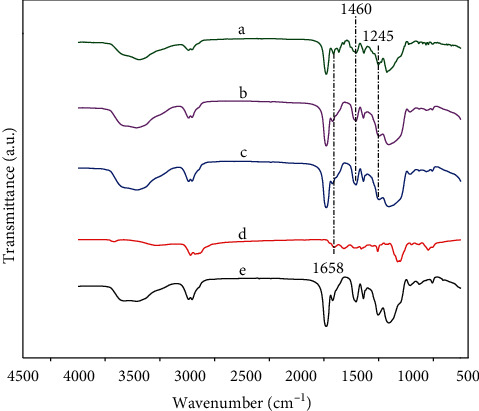
FT-IR spectra before (a) and after (b) extraction kaempferol of CDs@SBA-15@MIP. FT-IR spectra of CDs@SBA-15@NIP after extraction (c) as well as of CDs (d) and kaempferol (e).

FT-IR analysis of CDs@SBA-15@MIP and CDs@SBA-15@NIP composites showed similar patterns with characteristics bands of MIPs and NIPs at 1658, 1255, and 1460 cm^−1^ (Figure 1). Thus, structures and compositions of CDs@SBA-15@MIP and CDs@SBA-15@NIP were similar, and the only difference was that template molecules were extracted from MIPs during the imprinting process.

**Figure 2:**
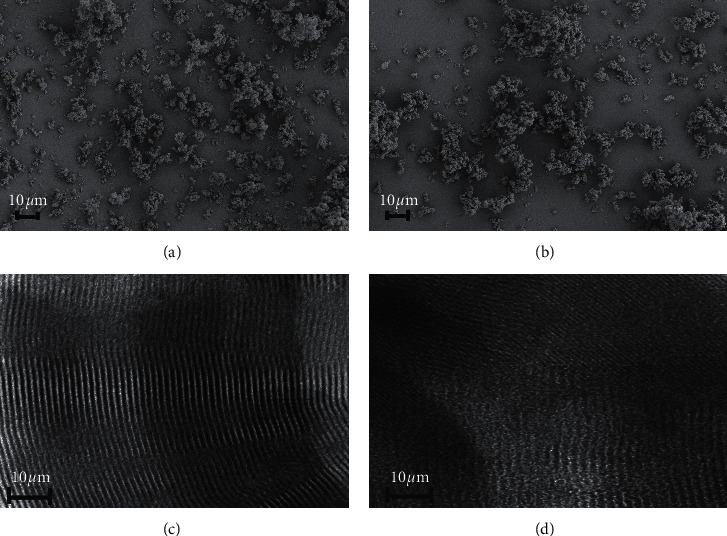
SEM micrographs images of (a) CDs@SBA-15-MIP and (b) CDs@SBA-15@NIP. TEM micrographs of (c) SBA-15 and (d) CDs@SBA-15@MIP.

Figures 2(a) and 2(b) show SEM images exhibiting the surface morphology of CDs@SBA-15@MIP and CDs@SBA-15@NIP. Both samples had a rough surface and a relatively narrow size distribution. The morphology of SBA-15 revealed via TEM indicated the presence of highly ordered hexagonal mesopore arrays aligned so as to form one-dimensional channels (Figure 2(c)). TEM of CDs@SBA-15@MIP also revealed a well-ordered morphology, in which the one-dimensional mesopore structure of SBA-15 was still visible (Figure 2(d)).

**Figure 3:**
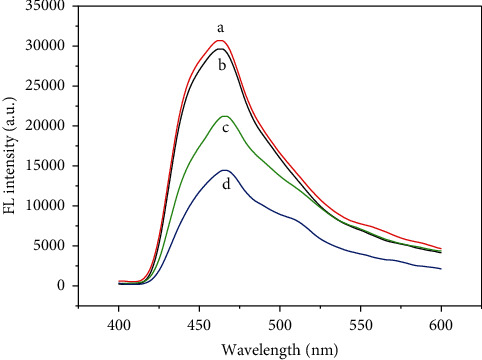
FL spectra of (a) CD_S_@SBA-15@MIP (after extraction), (b) CD_S_@SBA-15@NIP, (c) CD_S_@SBA-15@MIP (before extraction), and (d) CD_S_@SBA-15@MIP in the presence of 1.0 mg/L of kaempferol.

CDs@SBA-15@MIP was then used for the detection of kaempferol as shown in Figure 3. Fluorescence spectrum of CDs@SBA-15@MIP significantly changed upon kaempferol presence in the matrix judging by its significantly decreased intensity (Figure 3(c)), which decreased more at higher kaempferol concentration (Figure 3(d)). After the template was extracted, fluorescence intensity of CDs@SBA-15@MIP (Figure 3(a)) became similar to the intensity of fluorescence of CDs@SBA-15@NIP (Figure 3(b)). Furthermore, the degree of quenching is affected by the concentration of kaempferol.

**Figure 4:**
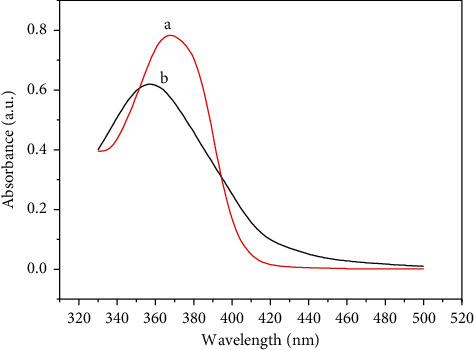
UV-vis spectra of kaempferol (a) and CDs@SBA-15@MIP (b).

Fluorescence quenching upon kaempferol presence might be because of energy resonance transfer (FRET), which occurs between two fluorescent molecules in proximity to each other. UV-vis spectrum of kaempferol and CDs@SBA-15@MIP has similar absorption band gap values (Figures 4(a) and 4(b), respectively). Thus, energy resonance transfer would be easy between these two molecules. When the UV absorption peaks of kaempferol and FL peak of CDs@SBA-15@MIP overlap, nonradiative energy transfer occurs [22].

**Figure 5:**
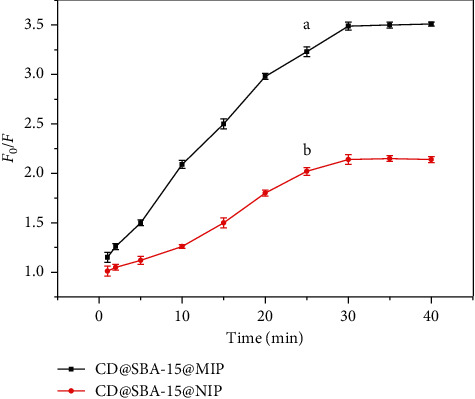
Kinetic uptake of kaempferol molecules by (a) CDs@SBA-15@MIP and (b) CDs@SBA-15@NIP.

Analysis of adsorption kinetics helped us to evaluate FL quenching response rate of MIP. Fluorescence quenching value (*F*_0_/*F*) of the MIPs and NIPs was studied at different adsorption times. Sensor based on CDs@SBA-15@MIP showed higher fluorescence response to kaempferol than sensor based on CDs@SBA-15@NIP because of higher cavitation density and specific recognition sites (Figure 5). Adsorption of CDs@SBA-15@MIP was fast, and adsorption balance was achieved within 30 minutes. Therefore, 30 min was selected as the best detection time. Materials synthesized by traditional bulk polymerization techniques usually demonstrate slower adsorption rates: sometimes up to 24 hours are needed to achieve equilibrium [23].

### 3.2. Specificity and Selectivity Experiments

**Figure 6:**
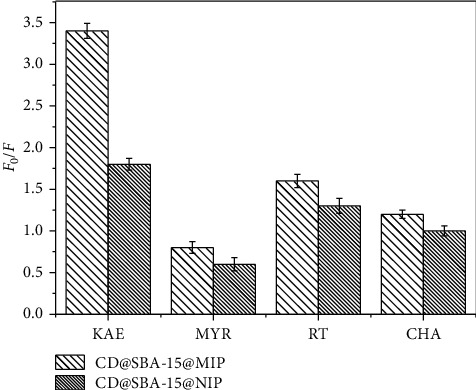
FL response of CDs@SBA-15@MIP and CDs@SBA-15@NIP towards kaempferol and its three structural analogues: CHA, MYR, and RT.

Specificity and selectivity are the key characteristics demonstrating how successful molecular imprinted polymers are. Thus, we studied sensitivity of CDs@SBA-15@MIP relative to kaempferol and its structural analogues (such as myricetin (MYR), rutin (RT), and chlorogenic acid (CHA). FL responses (presented as *F*_0_/*F*) of CDs@SBA-15@MIP and CDs@SBA-15@NIP to kaempferol (KAE), MYR, RT, and CHA are shown in Figure 6. FL response of CDs@SBA-15@MIP relative to 0.5 mg/L of kaempferol was much larger than for its structural analogues, which confirms better adsorption capacity. High specificity of CDs@SBA-15@MIP towards kaempferol was probably because shapes of MIP cavities fitted very well with kaempferol molecular structure. Other analogues did not bind strongly enough on the imprinted cavities.

**Figure 7:**
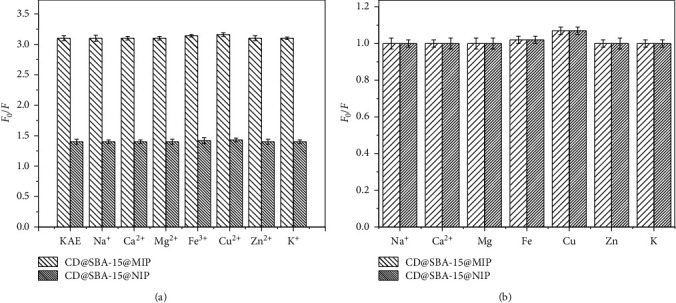
(a) Effect of the presence of metal ions on the recognition of kaempferol by CDs@SBA-15@MIP and CDs@SBA-15@NIP. (b) Identification of metal ions on CDs@SBA-15@MIP and CDs@SBA-15@NIP.

Metal ions can also cause quenching of carbon point fluorescence. To investigate resistance of CDs@SBA-15@MIP and CDs@SBA-15@NIP to interference from metal ions, fluorescence of CDs@SBA-15@MIP and CDs@SBA-15@NIP was measured under the presence of Na^+^, Ca^2+^, Mg^2+^, Fe^3+^, Cu^2+^, Zn^2+^, and K^+^. Binding capacity of CDs@SBA-15@MIP and CDs@SBA-15@NIP towards kaempferol in the presence of different metal ions is shown in Figure 7(a).  Figure 7(b) shows metal ions on CDs@SBA-15@MIP and CDs@SBA-15@NIP.

These results illustrate that metal ions did not change FL signals of CDs@SBA-15@MIP and CDs@SBA-15@NIP under the kaempferol presence. Statistical analysis showed no signification difference between the groups (*p* > 0.05). Thus, CDs@SBA-15@MIP seems to be an ideal sensor for the highly selective detection of kaempferol.

### 3.3. Kaempferol Detection Using Calibration Curve

**Figure 8:**
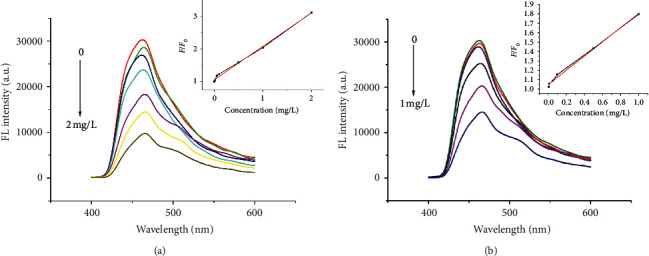
FL spectra of CDs@SBA-15@MIP (a) and CDs@SBA-15@NIP (b) in the presence of different kaempferol concentrations.

Changes in FL intensity of CDs@SBA-15@MIP and CDs@SBA-15@NIP after adsorption of different concentrations of kaempferol are shown in Figure 8.

FL intensity decreased significantly as kaempferol concentration increased. FL quenching of any system typically follows the Stern–Volmer equation [24]:

(1) $$\begin{array}{c} \frac{F_{0}}{F}=\text{Ksv}\left[Q\right]+1, \end{array}$$

where *F*_0_ and *F* are FL intensities in the absence and presence of kaempferol, respectively; Ksv is the Stern–Volmer constant, and [*Q*] is the quencher concentration. We used this equation to quantify various quenching constants as well as Ksv ratios for MIP and NIP (Ksv-MIP/Ksv-NIP), which was used to define imprinting factor to determine selectivity.

A linear relationship between FL response and kaempferol concentration from 0.05 to 2.0 mg/L showed correlation coefficient equal to 0.9973. The corresponding equation was as follows: *F*_0_/*F* = 1.016*Q* + 1.056.

Limit of detection (LOD) was 14 *μ*g/L which is equal to 3*σ*/*k*, where *σ* is standard deviation of the response and *k* is the intercept of the calibration curve.

### 3.4. Kaempferol Detection in Fruits and Vegetables Using CDs@SBA-15@MIPs

To test practical application of our sensor, seven fruits and vegetables samples were selected. To ensure accuracy, we selected samples with low concentration of kaempferol for standard recovery experiment to eliminate matrix interference [9, 25]. Three high concentrations of kaempferol were used to obtain sensor FL in order to later calculate its recovery rate. Recovery and RSD of kaempferol were 80–112% and 1.48–7.41%, respectively. These values show feasibility of the kaempferol detection method in actual food samples. FL and HPLC results are shown in Table 1.

Detection results of CDs@SBA-15@MIP as a sensing material with fluorescence response in all seven food samples agreed very well with the HPLC results. Thus, the method developed in this work demonstrated high accuracy and can be used for practical applications because it satisfies detection requirements for the actual food samples. Detection procedure of this method was simple, accurate, and fast. Thus, it has a strong potential to be widely used in variety of practical applications.

## 4. Conclusions

Composites based on MIPs containing CDs-embedded SBA-15 were fabricated using a simple, one-stage, and one-pot surface-imprinting synthesis technique. The resulting CDs@SBA-15@MIP material was used to fabricate sensor for fast and simple detection and concentration determination of kaempferol in food. SBA-15 used in this work had a well-defined mesoporous structure, which demonstrated excellent affinity and high capacity towards kaempferol as well as overall stability, all of which makes it an excellent adsorbent relative to kaempferol. Advantages of the sensor are reduced analysis time, excellent recovery, and repeatability, all of which eliminate complex multistep preparation of food samples required for equipment-intensive methods such as HPLC and MS. A method like ours offers rapid identification and quantification of a single component; thus, we strongly believe it has future in practical applications.

## Figures and Tables

**Table 1:** Recovery data for kaempferol detection in various food samples obtained using CDs@SBA-15@MIPs and HPLC.

Sample	Added (*μ*g·kg^−1^)	FL			HPLC		
		Found (*μ*g·kg^−1^)	Recovery (%, *n* = 3)	RSD (%)	Found (*μ*g·kg^−1^)	Recovery (%, *n* = 3)	RSD (%)
Coriander	5	4.7	94	3.47	5.6	112	7.5
	25	24.9	99.6	1.73	26.2	104.8	6.3
	50	50.1	100.2	2.48	44.6	89.2	5.5
Strawberry	5	4.9	98	5.22	3.5	90	15.1
	25	21.6	86.4	7.41	20	80	6.9
	50	48.4	96.8	3.98	47.5	95	5.8
Orange	5	4.4	88	1.48	4.6	92	13.7
	25	23.6	94.4	1.61	28.1	112.4	9.4
	50	52.4	104.8	1.48	45.4	90.8	6.8
Tomato	5	4.9	98	1.65	4.6	92	9.4
	25	23.9	95.6	3.59	26.8	107.2	4.8
	50	43.5	87	5.54	40.3	80.6	3.3
Carrot	5	4.6	92	1.91	4.4	88	13
	25	24.2	96.8	2.32	23.4	93.6	5
	50	51.4	102.8	2.8	44.2	88.4	6.7
Celery	5	4.4	88	3.57	4	80	12.8
	25	23.8	95.2	4.3	25.8	103.2	10.4
	50	45.9	91.8	7.34	46.9	93.8	5
Apple	5	4.3	86	2.3	3.7	82	12.7
	25	23.2	92.8	3.71	18.4	84	8.9
	50	49.4	98.8	2.2	49.7	99.4	6

## Data Availability

The data used to support the findings of this study are available from the corresponding author upon request.
